# Can race really be erased? A pre-registered replication study

**DOI:** 10.3389/fpsyg.2014.01035

**Published:** 2014-09-16

**Authors:** Wouter Voorspoels, Annelies Bartlema, Wolf Vanpaemel

**Affiliations:** Faculty of Psychology and Educational Sciences, University of LeuvenLeuven, Belgium

**Keywords:** replication, social categorization, cognitive processing, coalitional psychology, categorization

## Abstract

When encountering an unknown individual, social categorization of the individual has been shown to automatically proceed on the basis of three fundamental dimensions: People seem to mandatorily encode race, sex and age. In contradiction to this general finding, Kurzban et al. (2001) showed that race encoding is not automatic and inevitable, but rather a byproduct of categorization in terms of coalitions. In particular, they argue and empirically support that when other coalitional information is present, the encoding of race is spectacularly reduced. In the present contribution, we present a replication of the race-erased effect reported by Kurzban et al. First, we give a detailed overview of the hypotheses, the experimental methodology, the derivation of the sample size required to achieve a power of 95%, and the criteria that need to be met for a successful replication. Then we present the findings of an empirical test that met the requirements of our power analyses. Our results indicate that the encoding of race is indeed reduced when another coalitional cue is available, yet this reduction is less marked than in the original study. This experiment was preregistered before data collection at Open Science Framework, osf.io/vnhrm/.

## 1. Introduction

When encountering an unknown individual, social categorization of the individual has been shown to automatically proceed on the basis of three fundamental dimensions: People seem to mandatorily encode race, sex and age (e.g., Taylor et al., 1978; Brewer, 1988; Fiske and Neuberg, 1990; Hewstone et al., 1991; Stangor et al., 1992; Hamilton et al., 1994). In contradiction to this general finding, Kurzban et al. (2001) showed that race encoding is not automatic and inevitable, but rather a byproduct of categorization in terms of coalitions (see also Cosmides et al., 2003). In particular, they found that when coalitional cues are orthogonal to racial cues, the tendency to categorize people on the basis of their race is strongly reduced. Given the ever more present reality of multi-ethnic societies, this *race-erased effect*^1^ is of crucial societal relevance. Moreover, as it is essentially a finding on categorization, it is a tremendously valuable contribution that can be made from the perspective of cognitive psychology.

Despite the relevance of the race-erased effect and its high number of citations, whether the effect can be replicated. Admirably, Kurzban et al. (2001) themselves report data from a successful replication attempt. Recently, Johnson and Cesario (2013), however, failed to replicate the effect, although they did observe a trend in the expected direction. We have no knowledge of any other archived or non-archived direct replication of the race-erased effect.

In light of the ongoing discussion between advocates of automatic encoding of race and researchers claiming that no special role is played by race, the key findings by Kurzban et al. (2001) that form the object of our replication proposal are that race encoding can be decreased in coalitional contexts, and that arbitrary cues other than racial appearance can play a role similar to that of race in earlier claims of automatic race encoding. In particular, in cases where race is not a valid cue for coalition, an alternative cue that is valid to infer coalition will be encoded more strongly than race.

## 2. Methods

Kurzban et al. (2001) assesses race encoding by means of a memory confusion protocol, developed by Taylor et al. (1978). In a first phase, participants were asked to form an impression of persons that engage in a discussion. In particular, Kurzban et al. (2001) presented participants with a sequence of sentences paired with a photograph of an individual that made this statement. The individuals differed on (at least) two dimensions: their race, being either Euro-American or African-American, and their coalition, belonging to either of two rival basket teams. When participants were then asked to match the sentences (presented in random order) with the individuals that uttered them in the discussion, their mismatches reveal their encoding (racial or coalitional), as individuals that are similarly encoded are more easily confused.

In what follows, we give a more detailed description of the method that will be followed in the replication study.

### 2.1. Materials and procedure

For the presentation of the experiment, we will follow the set-up of the original experiment, which we describe below. Deviations from the set-up will be explicitly discussed later on.

The stimuli consist of a series of photographs and a sequence of sentences. Each photograph shows the head and upper body part of one of eight individuals against a neutral background. For each individual, there are two identical photographs, that differ only in t-shirt color, which is either gray or yellow. The sentences comprise 24 antagonistic statements in a heated conversation. The stimuli are available on osf.io/vnhrm/. The sentences are reproduced Appendix C in Supplementary Material.

In a first phase, participants are shown the sequence of sentences paired with the photographs of the eight individuals. They are told that they will witness individuals of two rival basketball teams that had been in a fight during the previous season, and that these individuals uttered the sentences in the context of a group discussion. The task for the participants is to form an impression of the individuals in the discussion. Each participant views all 24 sentences, one by one, for 8.5 s each. The order of the sentences is fixed, as they are antagonistic statements in a heated discussion. For each participant the two teams are constructed randomly, with the provision that the players are equally divided in terms of race, i.e., each team consists of two Euro-American and two African-American players. In linking the sentences to photographs, teams alternate sentences and players were randomly assigned to sentences, with the restriction that the first four sentences were uttered by two Euro-American players followed by two African-American players or two African-American players followed by two Euro-Americans.

After this initial phase, participants engage in a 1-min distractor task. In particular, participants are asked to think of as many of the 50 US states and their capitals as they can, assisted by presentation of a map of the USA on the display. In the test phase, an array of photographs of all eight individuals (in randomized order) is presented to the participants. One at a time, each of the 24 sentences of the discussion appears on the screen (also in randomized order), and the participants are asked to recall and indicate which individual uttered the sentence. For each sentence, a photograph has to be selected to be able to proceed with the experiment.

A document with screenshots and the general flow of the experiment can be found on osf link osf.io/vnhrm/ (see also Supplementary Material Appendix A).

#### 2.1.1. Experimental conditions

Kurzban et al. (2001) theorized that enhanced coalition information can boost coalitional encoding, to the expense of racial encoding. To test this prediction, they used two conditions (which they called experiments 1 and 2). The basic procedure, as described above, is identical in the two conditions, yet the salience of coalitional information varies. In the *no visual cue* condition, all eight individuals have the same shirt (either yellow or gray). Thus, there are no visual cues that reveal team membership in the discussion between the eight individuals, and only verbal cues can be used to infer team membership (e.g., “You were the ones that started the fight,” see Appendix C in Supplementary Material). The content of the sentences and the order in which they are uttered in principle contain sufficient information to infer which team an individual is on. In the *visual cue* condition, team membership is emphasized by means of colored t-shirts (either gray or yellow t-shirts, correlating perfectly with team membership) worn by the basketball players. Participants in the visual cue condition can infer team membership from both verbal cues and visual cues.

#### 2.1.2. Dependent variables

The errors people make, that is, the mismatches of a particular sentence and a photograph, form the basis of the data analysis. Four mismatches can be made: Confronted with a statement, a participant can erroneously select (1) another player from the same team and of the same race (one photograph matches this profile), (2) a player on the same team but of a different race (two photographs), (3) a player from a different team and of the same race (two photographs), or (4) a player from a different team and of a different race (two photographs). On the basis of the type of mismatches, two scores are derived for participants, denoting the extent to which they encoded coalition (that is, team membership) and the extent to which they encoded race.

Since the same-team same-race error has a lower prior probability than the other errors, the error rates of the other three types of errors are divided by two (Kurzban et al., 2001; Taylor et al., 1978). After this correction, the extent to which participant *i* encoded of the coalition (*t_ic_*) is assessed as follows:

(1) $$t_{ic}=(E_{i}^{cr}+E_{i}^{c\neg r})-(E_{i}^{\neg cr}+E_{i}^{\neg c\neg r})$$

with *E^cr^_i_* referring to participant *i*'s mismatches of the type same coalition and same race, *E^c¬r^_i_* referring to mismatches of the type same coalition and different race, and so on. Applying the same notation, encoding of the race dimension for participant *i* (*t_ir_*) is given by:

(2) $$t_{ir}=(E_{i}^{cr}+E_{i}^{\neg cr})-(E_{i}^{c\neg r}+E_{i}^{\neg c\neg r})$$

### 2.2. Differences from original study

We attempted to mimic the original experiment as closely as possible, in terms of materials used. We had available the experiment program, the instructions and the sentences in the discussion between the two teams from the original study (Kurzban, personal communication, June 2013). Due to unavailability of the original photographs, we used photographs from the replication by Johnson and Cesario (2013), and adapted them to resemble the original photographs (as shown in Cosmides et al., 2003 Figure 1) in terms of t-shirt color. Furthermore, in the original experiment photographs were of the same size in the discussion and the test phase (Kurzban, personal communication, April 2014). In the present experiment, the size was slightly smaller in the test phase.

Unlike Kurzban et al. (2001), we ran the experiment through Amazon's Mechanical Turk. As data provided by workers on Mechanical Turk can be improved by additional questions that have explicitly verifiable answers (Mason and Suri, 2012), we included three questions after presenting the general instructions, that require correct answering before being directed to the actual experiment. The questions were easy multiple choice questions, that should present no obstacle if the instructions were read and understood. If the questions were not correctly answered, participants were redirected to the instructions. If, on their second try, they did answer the questions correctly, they were able to proceed with the experiment. If not, they were not allowed to participate. The questions are provided Appendix B in Supplementary Material, as well as on osf.io/vnhrm/.

### 2.3. Recruitment

Participants were recruited using Amazon's Mechanical Turk. The experiment took about 12 min to complete, and participants were paid 2 USD, which is in line with a standard of 10 USD per hour. We restricted participation to USA-residents to optimize comparability with the original study. Sex ratio was monitored to be close to 50 : 50.

## 3. Confirmatory analysis plan

The original design of experiments 1 and 2 and the replication in 5 and 6 (Kurzban et al., 2001), can be conceived as a simple experimental set up with two between-subjects conditions: no visual cue and visual cue. To recapitulate, in a first phase participants witness a discussion between two teams, each represented by four members, presented by means of photographs and sentences. In one condition, the photographs do not contain visual cues as to team membership, and only verbal cues can be used to determine team membership. In the second condition, speakers can be easily recognized as members of either team through colored t-shirts. In the current replication proposal, we set out to test two crucial hypotheses, which directly map on predictions 3 and 4, in Kurzban et al. (2001):

- Hypothesis 1 (*ℋ*_1_): when visual coalition cues are present, the encoding of race is diminished as compared to when no visual coalitional cues are present.
- Hypothesis 2 (*ℋ*_2_): when visual coalition cues are present, the encoding of coalition is strengthened as compared to when no visual coalition cues are present.

### 3.1. Data cleaning plan

In Kurzban et al. (2001) no data filtering procedures were adopted (personal communcation, July 2013). In line with this, we did not exclude data points from the data set on the basis of data profile. We piloted the experiment with five participants on Mechanical Turk to ensure its functionality. Data from these test runs were not used. Apart from the testruns, we excluded data on the basis of the following three criteria, which were established before data collection: First, if the same participant (i.e., with the same MT worker ID) participated twice, only the data from the first participation was included. Second, participants that failed twice at answering the pre-test questions, were not allowed to perform the experiment and thus did not provide data. Finally, incomplete experiment runs, due to stopping the experiment for whatever reason before ending, were not considered.

### 3.2. Quantifying the hypotheses

Within each condition, participant *i* has a score for the encoding of race (*t_ir_*) as well as a score for the encoding of coalition (*t_ic_*). If we refer to the condition without visual cues as “0” and the condition with visual cues as “1,” the two hypotheses can be formulated as follows:

(3) $$\begin{array}{l} {ℋ}_{1}:\mu_{t_{r0}}>\mu_{t_{r1}}\\ {ℋ}_{2}:\mu_{t_{c0}}<\mu_{t_{c1}} \end{array}$$

with μ_*t*_*r*1__ referring to the mean dimensional coding score for race for condition 0 (that is, no visual cues), and so on. Both hypotheses will be tested using one-sided independent-samples *t*-tests, following Kurzban et al. (2001).

### 3.3. Sampling plan

The sampling plan is based on a power analysis considering all information available to us regarding direct replication attempts of the race erased effect, that is the report of four experiments in Kurzban et al. (2001) and one experiment reported in the conference presentation of personal communication, june 2013 (Johnson and Cesario, 2013)^2^. All relevant available information for *ℋ*_1_ and *ℋ*_2_ is presented in Table 1, presenting the sample sizes (*n*), means (*M*), standard deviations (*SD*), t-statistics (*t*), reported effect sizes *r* and Cohens d (*d_z_*) for all three experiments under consideration. The R-code for the calculations in this section can be found on OSF osf.io/vnhrm/.

**Table 1:** **Available summary statistics regarding *ℋ*_1_ and *ℋ*_2_ for all known studies**.

	**Study**	**No visual cue**			**Visual cue**			**Comparison statistics**		
		***n***	***M***	***SD***	***n***	***M***	***SD***	***t***	***r***	***d_z_***
*ℋ*_1_	Kurzban A	55	2.3	2.76	52	1.4	2.51	1.77	0.18	0.37
	Kurzban B	51	1.86	2.69	52	0.37	2.45	2.94	0.28	0.58
	J and C	90	1.84	2.59	85	1.44	2.64	1.01	0.08	0.16
*ℋ*_2_	Kurzban A	55	0.9	2.76	52	4.62	3.62	5.95	0.52	1.22
	Kurzban B	51	na	na	52	na	na	na	na	na
	J and C	90	na	na	85	na	na	na	na	na

*“Kurzban A” refers to experiments 1 and 2 and “Kurzban B” refers to experiments 5 and 6 in Kurzban et al. (2001). “J and C” refers to the study reported in Johnson and Cesario (2013). All values except *t* and *d_z_* are taken from the published paper, or were provided by the authors. The t-statistics were calculated with $t=M_{1}-M_{2}/\sqrt{sd_{1}{}^{2}/n_{1}+sd_{2}{}^{2}/n_{2}}$. The effect size *d_z_* was computed using Equation 4. As indicated in the main text, we did not have sufficient information to fill in a number of cells (na)*.

While all studies under consideration reported *r*, most tools for computing sample size rely on *d_z_* as a measure of effect size. We transformed the reported effect size *r* to the effect size *d_z_* using the following equation (Rosnow et al., 2000, combining their Equations 7, 11, and 15):

(4) $$d_{z}=\frac{\left(n_{1}+n_{2}\right)r}{\sqrt{n_{1}n_{2}\left(1-r^{2}\right)}}$$

in which *n_1_* and *n_2_* refer to the sample sizes of the two conditions in the experiments, and *r* refers to the reported correlation effect size^3^.

After deriving *d_z_* for the relevant effects, we pooled the effect sizes for *ℋ*_1_ across the three experiments by weighting each individual effect size with its inverse variance (Cooper et al., 2009):

(6) $$\bar{d_{z}}=\frac{\sum_{i=1}^{k}w_{i}{\hat{d}}_{zi}}{\sum_{i=1}^{k}w_{i}},$$

where *k* is the number of experiments and *w_i_* is the inverse of the variance of effect size $\hat{d}$_*zi*_ in experiment *i*:

(7) $$w_{i}=\frac{1}{\frac{n_{i1}+n_{i2}}{n_{i1}n_{i2}}+\frac{d_{zi}^{2}}{2\left(n_{i1}+n_{i2}\right)}},$$

where *n*_*i*1_ and *n*_*i*2_ are sample sizes of the two groups in experiment *i*.

The pooled effect size of the effect for *ℋ*_1_ is is 0.328. For *ℋ*_2_, we do not have information over and above experiments 1 and 2 reported in Kurzban et al. (2001), so the pooled effect size equals the single available effect size 1.218. Using the pwr-package in R, we calculated a planned sample size for achieving a 0.95 power level using a one-sided independent-samples *t*-test. This resulted in a sample size of 202 per condition for *ℋ*_1_, and thus in total 404 participants. For *ℋ*_2_, a sample size of 15 per condition would be sufficient. To achieve maximal power, we used the largest of these two.

### 3.4. Replication criterion

Our attempt to replicate (Kurzban et al., 2001) can be deemed successful if both hypotheses are confirmed by the data. The following protocol was established prior to data collection: For *ℋ*_1_ we perform a one-sided independent samples *t*-test comparing racial encoding in the visual-cue condition to racial encoding in the no-visual cue condition, and we reject the null hypothesis that there is no difference if the *p*-value is smaller than 0.05. For *ℋ*_2_ we perform a one-sided independent samples *t*-test comparing coalitional encoding in the visual cue condition to coalitional encoding in the no-visual cue condition, and we reject the null hypothesis that there is no difference if the *p*-value is smaller than 0.05. We also provide information regarding the effect sizes (and standard errors) for each of the effects tested. The specification of this protocol prior to data collection can be verified on OSF-link osf.io/vnhrm/.

## 4. Results

The raw and post-processed data are available on the Open Science Framework at osf.io/vnhrm/, together with the R code R Core Team (2013) for the sample size calculation, the post-processing of the data and the analyses presented below.

### 4.1. Sample

The link to our experiment was clicked 876 times and 503 hits resulted in initiating the experiment. Of these visitors, 460 unique participants completed the entire experiment, and three finished the experiment but did not enter their worker's ID (which was asked in the debriefing, after completing the actual experiment). Although strictly, these three participants have an incomplete run, they did complete the actual experiment, and thus their data were not excluded ^4^.

Of the 463 participants that completed the experiment, 225 were assigned to the no visual cue condition and 238 to the visual cue condition. The average age in the sample was 33.36, and the gender ratio 45 : 55 (females and males respectively). The majority of the participants indicated to be Caucasian (78%), followed at a distance by African (9%) and Asian (6%). All participants indicated they resided in the USA (this was also a requirement for participation in the experiment). As can be expected, chi-square tests did not refute independency between condition assignment and any of the demographic variables. Table 2 provides more detailed information on the sample.

**Table 2:** **Demographic information of the sample**.

**Condition**	***n***	**Gender**	**Age (*SD*)**	**Ethnic background**			
				**Caucasian (%)**	**African (%)**	**Asian (%)**	**Latino (%)**
No visual cue	225	46:54	33.25 (10.92)	78	11	5	3
Visual cue	238	45:55	33.47 (10.58)	78	7	6	3
Total	463	45:55	33.36 (10.73)	78	9	6	3

### 4.2. Confirmatory analyses

**Figure 1:**
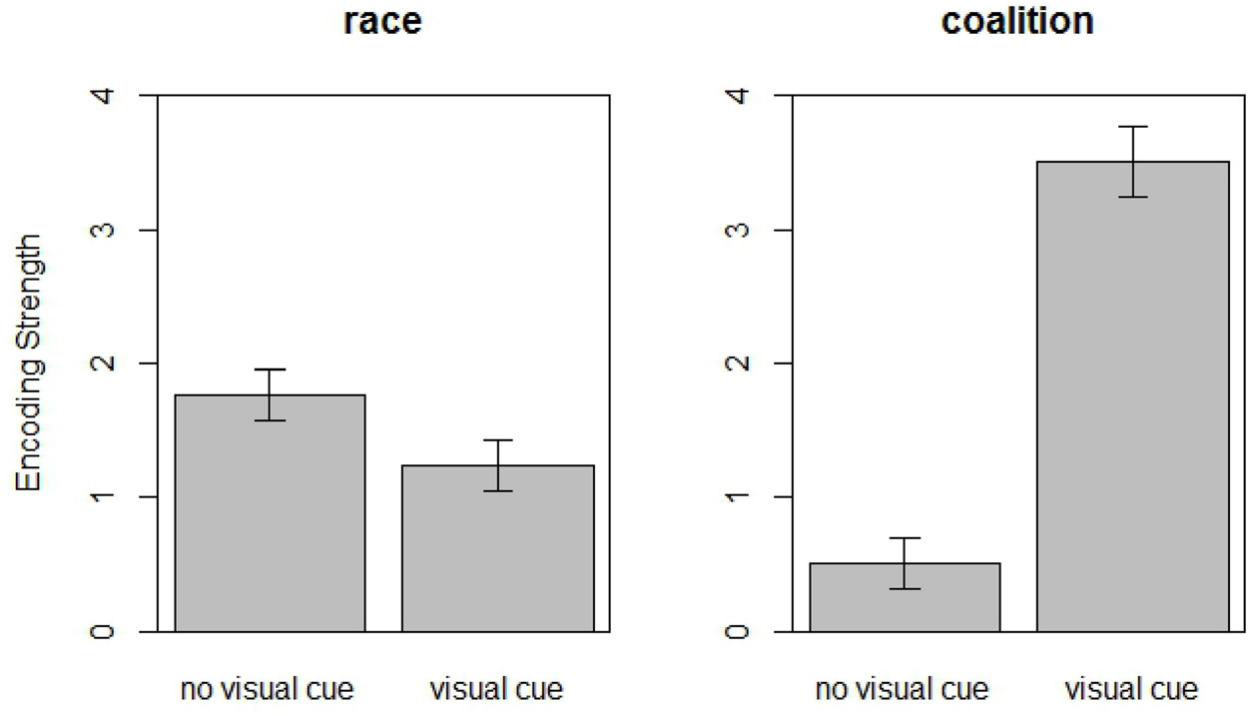
**Means and standard errors of race encoding and coalition encoding as a function of condition**.

Two hypothesis tests were planned, both regarding differences between the condition in which only verbal utterances are indicative of team membership (*no visual cue*) and the condition in which differently colored t-shirts are also indicative of team membership (*visual cue*). Figure 1 presents the means of encoding of race (left graph) and encoding of coalition (right graph) in the two conditions. For more details, we refer to Table 3.

**Table 3:** **Summary statistics regarding *ℋ*_1_ and *ℋ*_2_, for the current study**.

	**No visual cue**			**Visual cue**			**Comparison statistics**			
	***n***	***M***	***SD***	***n***	***M***	***SD***	***t***	***p***	***r***	***d_z_***
*ℋ*_1_	225	1.77	2.85	238	1.24	2.89	1.99	0.023	0.09	0.185
*ℋ*_2_	225	0.50	2.78	238	3.50	4.10	9.26	<0.001	0.39	0.852

First, does the encoding of race decrease when a clear visual coalitional cue is available (*ℋ*_1_)? We performed an independent-samples, one-sided *t*-test, comparing the encoding of race in the no visual cue and visual cue conditions. The test revealed a significant difference [*t*_(461)_ = 1.99, *p* = 0.023, *r* = 0.09], rejecting the null hypothesis associated with *ℋ*_1_. The effect size, *r* = 0.09, is smaller than the effect sizes reported in (experiments 1 and 2: *r* = 0.18 and experiments 5 and 6: *r* = 0.28) (Kurzban et al., 2001), and on par with the effect size reported by (*r* = 0.08) (Johnson and Cesario, 2013), as can be found in Table 1. Moreover, the confidence interval on the effect size only just excludes 0 [95%CI (0.002, 0.182)].

The second hypothesis concerns an increase in encoding of coalition when the visual cue is present (*ℋ*_2_). Again, an independent-samples, one-sided *t*-test, comparing the encoding of coalition across the two conditions, was performed. The test revealed a significant difference [*t*_(461)_ = 9.26, *p* < 0.001, *r* = 0.39), rejecting the null hypothesis associated with *ℋ*_2_. Again, the effect size is smaller than the effect size in Kurzban et al. (2001). The confidence interval on the effect size is well above 0 [95%CI (0.31, 0.46)].

**Figure 2:**
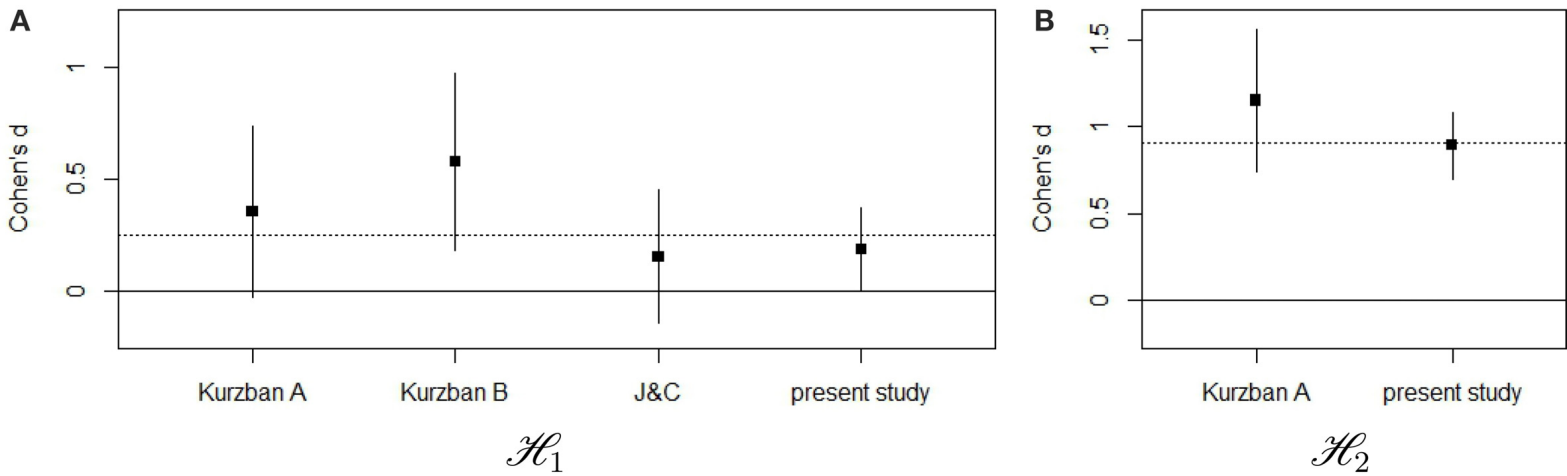
**Comparison of the effect sizes (Cohen's d) across different studies for *ℋ*_1_ (A) and *ℋ*_2_ (B)**. Black squares represent the point estimates for the respective Cohen's d, and the associated vertical lines demarcate the 95% confidence interval. The dotted horizontal line represents the pooled effect size across all studies.

In sum, our planned analyses confirmed the results of Kurzban et al. (2001) for both *ℋ*_1_ and *ℋ*_2_, suggesting that the availability of a visual coalitional cue increases categorization on the basis of coalition and reduces categorization on the basis of race. Yet, it is important to recognize that the effect size of the reduction in race encoding was considerably smaller than the effect sizes reported by (experiments 1, 2, 5, and 6) (Kurzban et al., 2001) and the pooled effect size on which our sample size calculation was based. An overview of the effect sizes (Cohen's d) and confidence intervals for all studies available to us for both hypotheses can be found in Figure 2. Combining all effect sizes from the previous studies and the current study, we arrived at an estimated effect size of 0.25 for *ℋ*_1_, and 0.91 for *ℋ*_2_, which are shown by the dotted vertical lines in Figure 2.

### 4.3. Additional analyses

Following Kurzban et al. (2001), we performed paired *t*-tests between rates of different types of errors, testing whether within class errors were made more often than between class errors within condition^5^. Essentially, these tests evaluate, within each condition separately, whether the average of *t_ic_* and *t_ir_* (see Equations 1, 2) are significantly different from zero. Kurzban et al. (2001) considered the effect size of these tests to be a measure of the extent to which race (*t_ir_*) or coalition (*t_ic_*) is encoded. Table 4 presents the within race, between race, within coalition and between coalition errors in each condition, as well as effect sizes for the paired *t*-tests within conditions.

**Table 4:** **Average and standard deviation of the within class (wc) and between class (bc) errors in both conditions and effec sizes of the paired *t*-tests (*r*)**.

**Condition**	**Race**			**Coalition**		
	**wc errors**	**bc errors**	***r***	**wc errors**	**bc errors**	***r***
No visual cue	6.20 (1.97)	4.43 (1.48)	0.53	5.57 (1.86)	5.07 (1.56)	0.18
Visual cue	5.78 (2.05)	4.55 (1.52)	0.40	6.92 (2.42)	4.47 (2.22)	0.65

When no visual cue was present, participants made more within coalition errors than between coalition errors [*M* = 0.5, *t*_(224)_ = 2.73, *p* = 0.003], as well as more within race errors than between race errors [*M* = 1.77, *t*_(224)_ = 9.33, *p* < 0.001]. When a visual cue for team membership was available, participants made significantly more within coalition errors than between coalition errors [*M* = 3.50, *t*_(237)_ = 9.33, *p* < 0.001], as well as more within race errors than between race errors [*M* = 1.24, *t*_(237)_ = 6.62, *p* < 0.001].

**Figure 3:**
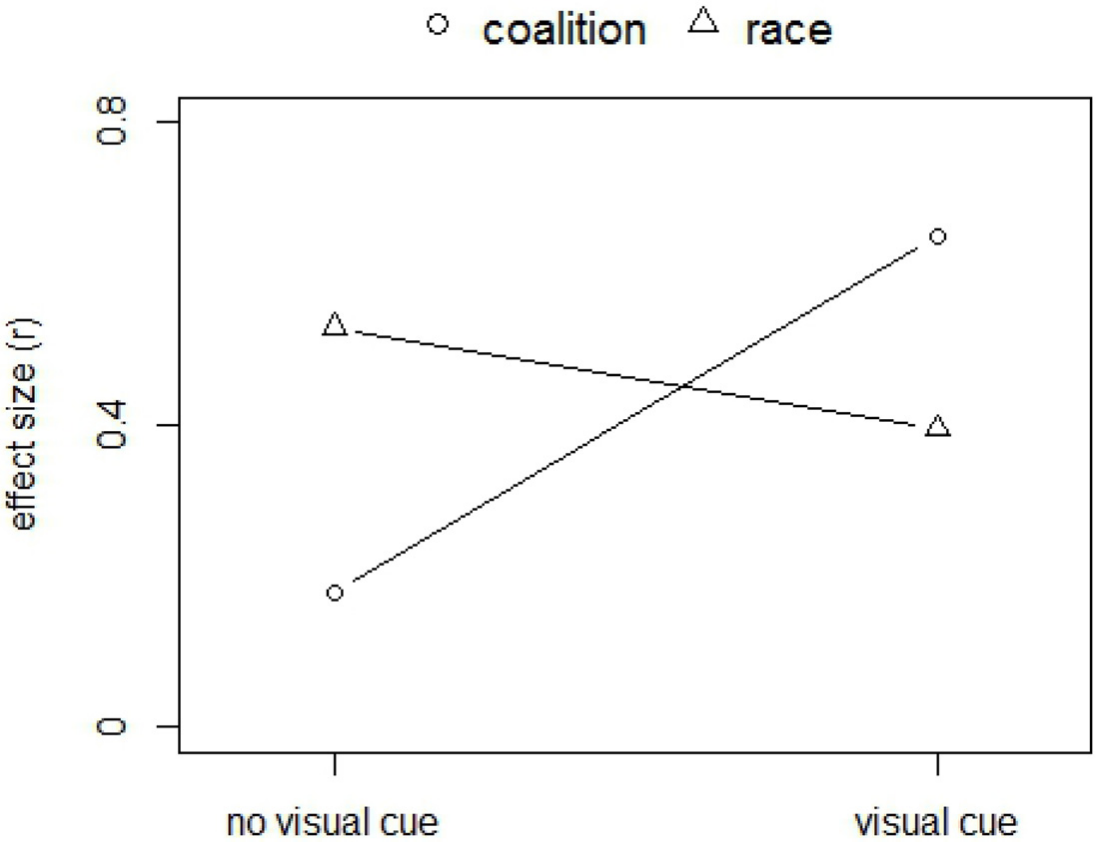
**Effect sizes (r) of the paired t-tests, comparing within class errors with between class errors, where class refers to either race or coalition**. Higher effect sizes suggest higher encoding of the respective class. This figure is comparable to Figure 2 of Kurzban et al. (2001).

In terms of effect sizes, a pattern qualitatively similar to that reported in Kurzban et al. (2001) emerged, as can be seen in Figure 3. In the no-visual-cue condition, the effect size of race is larger than the effect size of coalition [0.53, 95%CI (0.44, 0.61) vs. 0.18, 95%CI (0.05, 0.30), respectively]. Conversely, in the visual cue condition, the effect size of race is smaller than that of coalition [0.40, 95%CI (0.29, 0.47) vs. 0.65, 95%CI (0.57, 0.71), respectively]. Note that the confidence intervals on the effect size of race in the no-visual-cue and visual-cue condition slightly overlap.

In sum, although these additional analyses do not provide a direct test of the crucial hypotheses, they are consistent with the earlier tests and the pattern we observed qualitatively resembles the pattern reported in Kurzban et al. (2001). Notably, in comparison to the original study the effect size of race encoding decreases less markedly when the visual cue is available, which is consistent with our earlier analysis regarding *ℋ*_1_.

### 4.4. Bayesian data analysis

From a Bayesian data analytic perspective, we are interested in quantifying the evidence in favor of the hypotheses of interest, given the observations. An elegant way to do so involves calculating the Bayes factor, which weights the evidence for the hypothesis of interest against the evidence for the null hypothesis. This allows quantifying evidence for the alternative as well as for the null (contrary to classical null hypothesis testing). Bayes Factors were computed using the Bayes Factor package (Morey and Rouder, 2014).

For *ℋ*_1_—specifying that race encoding decreases—compared to the null hypothesis that there is no difference in race encoding between conditions, the Bayes factor suggested slight evidence in favor of *ℋ*_1_ (BF = 1.82). The Bayes factor indicates that *ℋ*_1_ is 1.82 times more likely than the null hypothesis, which is conventionally labeled as “anecdotal evidence.” As to the increase in coalitional encoding (*ℋ*_2_), the Bayes factor was overwhelmingly in favor (BF = 5.33*e*^15^)^6^. These results are qualitatively identical to Kurzban et al. (2001), but as in our other analyses, the evidence for a reduction in race encoding was rather weak.

## 5. Discussion and conclusion

Our replication qualitatively supports the results reported by Kurzban et al. (2001). On the basis of the original report and information from a recent similar study (Johnson and Cesario, 2013) we estimated that a sample size of at least 202 for each condition was required to arrive at a power of 0.95 for the crucial hypotheses, setting the criterion at 0.05 for a one-sided *t*-test. Our sample exceeded this requirement and the experimental setup mimicked—as closely as possible—the original experiment.

The planned *t*-tests confirmed the crucial hypotheses: We found that, in the context of a memory confusion protocol, the encoding of an individual's race decreases when a visual cue of team membership is introduced, and that the encoding of coalition increases when introducing such a cue. These findings were further supported by additional analyses of effect sizes and Bayesian *t*-tests. We did not, however, find support for the thesis that the encoding of race is entirely erased in a coalitional context. Indeed, the effect size for the reduction in race encoding was substantially lower than in the original study, and consequently, a Bayesian *t*-test revealed only “anecdotal evidence” in favor of a reduction effect.

## Author note

Wouter Voorspoels is a postdoctoral researcher at the Research Foundation—Flanders. Annelies Bartlema is a doctoral student funded by the Research Foundation—Flanders. Wolf Vanpaemel is an assistant professor at KU Leuven. We thank Lotte Dejaeghere and Kristof Meers for help with the construction and programming of the experiment and the data collection. We also thank Robert Kurzban, David Johnson, Joseph Cesario, and David Pietraszewski for their willingness to share materials and information, as well as comments and suggestions to improve the manuscript.

### Conflict of interest statement

The authors declare that the research was conducted in the absence of any commercial or financial relationships that could be construed as a potential conflict of interest.

## Footnotes

^1^It is, perhaps, more appropriate to speak of a *race-reduced* effect. We will, however, follow the terminology of the original paper.

^2^For the present purposes, we only use a subset of the data reported in Johnson and Cesario (2013). This subset maps onto the most direct replication in the study (Johnson, personal communication, June, 2013). Note that the use of the entire data set resulted in a larger effect size, and as such, the selection of data made our estimate of the required sample size more conservative.

^3^When *n*_1_ = *n*_2_, Equation 4 reduces to the more familiar expression :

(5) $$d_{z}=\frac{2r}{\sqrt{1-r^{2}}}.$$

^4^We also performed analyses excluding these participants, and this did not change results.

^5^A within class error occurs when one attributes a sentence to another individual of the same team (when the class of interest is team) or of the same race (when the class of interest is race). Between class errors occur when one attributes an utterance to an individual of a different team or a different race.

^6^We used the equivalent of a one-sided *t*-test, that is, the Bayes factors weight the evidence that the effect size is positive against the evidence that it is null. Since we had prior knowledge that the effect size for *ℋ*_2_ is relatively small (we estimated *d* to be 0.328), the prior on effect size was a Cauchy scaled by 0.5, as recommended by Rouder et al. (2009). Due to neither the null nor the alternative hypothesis being strongly supported by the data, the Bayes factor is closer to 1 for larger values of *r* (corresponding to larger a priori expected effect sizes): if *r* is set to $\sqrt{2}/2$, BF = 1.37 and if *r* is set to 1, BF = 1.01. For *ℋ*_2_, the prior was a Cauchy distribution scaled by 1, as effect size was expected to be substantially larger (*d* = 1.218).

## Supplementary material

Appendix A contains the flow chart of the experiment and the instructions. The instructions are identical to the ones used in Kurzban et al. (2001, personal communication, June 2013). The original experiment did not include items for the instruction check. Appendix B shows the pre-test questions. No pre-test questions were used in the original experiment. Appendix C shows the sentences, which are taken from Kurzban et al. (2001, personal communication, June 2013). Appendix D shows the informed consent, demographic questions, and debriefing. These were not taken from the original experiment.
