# Supplementary material for: Can race really be erased? A pre-registered replication study
Source: Front Psychol. 2014 Sep 16;5:1035. doi: 10.3389/fpsyg.2014.01035 (PMC4165323; doi:10.3389/fpsyg.2014.01035)
Supplement: Supplementary file 1 [file Presentation1.PDF]

Appendix A
Flow chart and instructions

[**INFORMED CONSENT**]

[**DEMOGRAPHIC INFO**]

[**INSTRUCTIONS**]

A number of years ago, there was an unfortunate incident involving two co-ed college basketball teams, one of which had a reputation for playing rough. Towards the end of the third period, tempers were running high. The score was extremely close, and there had been a history of rivalry between the two teams. The game had been what sports announcers usually refer to as 'extremely physical'. At one point, one of the players from the home team went down. The referees hadn't seen what had happened, but some players claimed to have seen a member of the visitor's team hit the guy as he was making a move toward the basket. In a rush, the home team's bench emptied, and a fistfight broke out. It took security several minutes to restore order, and in the end, another player was injured in the scuffle.

As punishment for the incident, both teams were put on probation. Far from settling the issue, this led to hard feelings on both sides. However, the teams' schedule calls for them to play each year, and so, next season, they met again. As part of an attempt at reconciliation, a number of players from each team were put together to talk before the game. This conversation was recorded, and you will be seeing a portion of it.

We are interested in the impressions that these players make on you as they have their discussion. You will see their photographs paired with what they said. Pay attention to the players because you will be asked to give your impressions of them after you have seen the discussion.

The photos you will be seeing will advance automatically, without your having to press anything. After you have completed viewing the photos and statements, you will receive further instructions on the computer screen. Click on ‘NEXT’ to begin.

[**INSTRUCTION CHECK**]

[**TEAM DISCUSSION IS SHOWN**]

[**FILLER TASK: INSTRUCTIONS**]

You have now seen all of the photos. We will now ask you to try to think of the names of as many of the fifty states and their capitals as you can. There is no need to write them down or type them into the computer. Simply try to think of them silently.

After a short period of time, you will receive further instructions. You may use the map of the U.S. to help you. Please click on ‘NEXT’ now.

[**U.S. MAP (ONE MINUTE)**]

[**SENTENCE-PHOTOGRAPH MATCHING TASK: INSTRUCTIONS**]

You are about to see all of the photos of the individuals that you saw in the first part of the experiment. In addition, you will see each statement made by the participants. Your task is to remember which person made each statement as you see it. Indicate your selection by pointing to the person’s photo and clicking once with the mouse.

It is very important that you give an answer for all of the comments. If you do not remember, try to guess. You may now click on ’NEXT’ to continue.

[**SENTENCE-PHOTOGRAPH MATCHING TASK**]

[**DEBRIEFING**]

Appendix B

Instruction check

Please first answer the following short questions:

You will be seeing a number of photographs of players. What sport do they play?

- football
- basketball
- water polo

By means of the photographs, you will witness:

- the players on a trip to the museum
- a conversation between the players
- a sport game in action

What do we want you to do?

- explain the game's rules
- judge the player's sport skills
- form an impression of the players

Appendix C

Team discussion

T1: “You guys started the whole thing. That was the most flagrant foul I’ve ever seen. Your guy should have been ejected for that.”

T2: “That’s bullshit. You have to play the whistle. No whistle, no foul.”

T1: “Hey, come on. He basically threw his elbow right into his face. You don’t pull stuff like that when you’re in our house.”

T2: “Hey, you were the ones that started the fight. This whole thing wouldn’t have happened if you could control yourselves.”

T1: “You nail our guy in the face and expect to just get away with it? No way.”

T2: “No one nailed anyone. It was a clean play. You guys get all bent out of shape, and now we’re both screwed. Thanks for blowing our whole season.”

T1: “Give me a break. We didn’t blow your season. You did. Let’s be serious.”

T2: “Look, the point is you got out of control, went nuts, and got us on probation too. It’s ridiculous.”

T1: “If you just played like civilized people, the whole thing would never have happened.”

T2: “You have to be kidding me. At least we don’t play like you. You play like you’re in high school.”

T1: “And you play like you’re in a zoo. Where you should be anyway.”

T2: “Look, you’re just a bunch of wimps. You were sore because we beat you.”

T1: “Yeah. After you took out our best guy by punching his lights out.”

T2: “You guys sure complain a lot. You should do more playing and less whining.”

T1: “Just shut up, man. You know the whole thing was your fault. You’re a

bunch of animals."

T2: "Hey, yo, you better watch it. You need to cool off, friend."

T1: "Don't be talking to us that way. You're asking for some serious trouble."

T2: "Yeah? And who's going to be making that trouble? You and the rest of the ladies here?"

T1: "The only reason you're not flat on your back right now is I don't want to get kicked out before the game even starts."

T2: "Oh, now we're really scared. What's the matter with you people? You just can't take it."

T1: "That's about enough. Don't you guys have something to do? In fact, why don't you get back on the bus."

T2: "We'll be leaving once we've gotten through with you. We'll see who's talking big after the game."

T1: "Well, if you guys won't leave. We will. Come on, we're out of here."

T2: "Fine. You guys take off. We'll see you on the court."

Appendix D

Informed consent, demographics and debriefing

Informed consent

Dear participant,

We constructed the following experiment to examine how people form impressions of other people. In particular, you will be asked to form an impression of a number of people engaging in a conversation. The research is done by Prof. Dr. Wolf Vanpaemel, Dr. Wouter Voorspoels, Annelies Bartlema & Lotte De Jaeghere (University of Leuven, Belgium). Before you start the experiment, please carefully read the following statements. If you have understood and agree with the statements presented below, please indicate so by clicking on the button “Yes, I agree”.

- I understand that the purpose of this study is to examine how people form impressions of other people.
- I am aware that there are no risks involved in participating in this study.
- I may benefit from this study in the following way: I will be compensated 2 USD for participating in this study.
- I am participating on a voluntary basis.
- The results of this study can be used for scientific purposes and can be published.
- My name will not be published and confidentiality of the data is maintained at each stage of the research.
- For any questions, complaints or further information, I know that I can contact:

- wolf.vanpaemel@ppw.kuleuven.be
- wouter.voorspoels@ppw.kuleuven.be

Demographics

- Are you male or female?
- How old are you?
- What country do you live in?
- What is your ethnicity?

- Caucasian
- Latino/Hispanic
- Middle Eastern
- African
- Caribbean
- Asian
- Mixed
- Other

- What is your mother tongue?

Debriefing

Dear participant,
Thank you for participating in this experiment! In the course of the experiment you were unexpectedly asked to reconstruct the conversation by linking sentences to photographs. You may have the feeling that you could have performed better if we had announced this task. However, we are interested in automatic processes that are involved when forming an impression of people. If we would have told you in advance that you had to reconstruct the conversation, conscious memorizing would have interfered with these processes, and would have distorted our results.

After the study is finished and analyzed, the results will be sent to you, if you provide us with your email address (note that this address will only be used for sending the results; the data will be analyzed anonymously). If you have further questions, don't hesitate to contact us: wouter. voorspoels@ppw.kuleuven.be

Pleas enter you Worker ID

Note: In order to be able to compensate you for your time, you need to do two things. First enter your worker ID in the box below. Second, after this window is closed, go back to the Amazon MTurk website and enter your worker ID below the description of this experiment.
